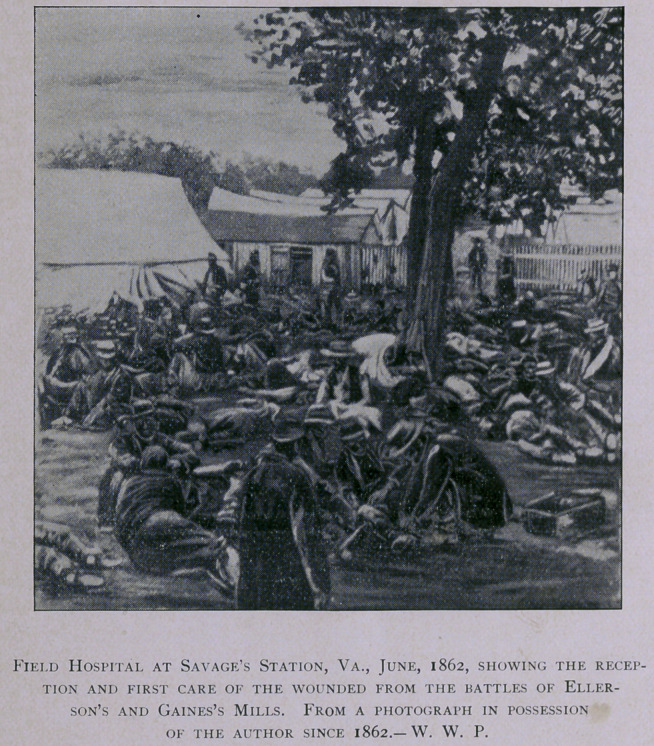# Reminiscences of Field-Hospital Service with the Army of the Potomac

**Published:** 1889-10

**Authors:** William Warren Potter

**Affiliations:** Brevet Lieutenant-Colonel United States Volunteers; Surgeon in Charge First Division Field Hospital, Second Army Corps; Surgeon Fifty-seventh Regiment, New York Volunteers; Assistant Surgeon Forty-ninth Regiment, New York Volunteers; Recorder Second Division Hospital in Sixth Army Corps, etc., etc.


Buffalo Medical and Surgical Journal

Vol. XXIX. OCTOBER, 1889. No. 3.

### Original Communications.

### REMINISCENCES OF FIELD-HOSPITAL SERVICE WITH THE ARMY OF THE POTOMAC.

By WILLIAM WARREN POTTER, M. D.,

Brevet Lieutenant-Colonel United States Volunteers; Surgeon in Charge First Division Field Hospital, Second Army Corps; Surgeon Fifty-seventh Regiment, New York Volunteers; Assistant Surgeon Forty-ninth Regiment, New York Volunteers; Recorder Second Division Hospital in Sixth Army Corps, etc., etc.

It is the purpose of the writer in these pages, kindly allotted to the consideration of this interesting phase of army life, to give a succinct account of the field-hospital system of the Army of the Potomac, based upon his experiences of three years' service as a medical officer in that army. Minute detail cannot, of course, be entered into within the necessarily narrow limits of a magazine article,—only distinctive features grouped and portrayed in outline.

If it were necessary to seek a ráison d'être for the appearance of such an article at this time, when so much is being written about the war and its conduct, it could be readily found in the fact that, so far, only officers of the line have figured in conspicuous prominence, as having achieved renown in the military service. It is an undeniable fact that the medical department of the army was very near the hearts of the millions of patriotic people who, while compelled to remain at home, contributed, with lavish hands, their means and substance toward the successful prosecution of the war. It is presumed that many of these will be interested to know something more of the manner of caring for the sick and wounded, in active service and on the field, that can be gleaned from ordinary or even official sources.

The writer served in the various capacities of Assistant Surgeon, and Surgeon on duty with the troops, and as Recorder of the Second Division Hospital, in the Sixth Army Corps; also, as assistant to Chief Operator, as Chief Operator, and as Surgeon-in-Charge of a division field hospital in the Second Army Corps, holding the latter place for more than a year. This statement is made that his opportunities for knowledge as to the working of the system may be understood, and the value of his judgment thereupon properly estimated.

An army in the field is, at once, confronted with the difficult problem of properly caring for its sick and wounded—a question second only in importance to the ever-present one of feeding it. The difficulties increase in a manifold degree if, as was chiefly the case with the Army of the Potomac, the field of operations lies in an enemy's country. Military reasons demand that disabled soldiers shall not impede the mobility of the columns; humane reasons insist, with equally cogent force, that they shall receive prompt and efficient care, and these with due regard to economy of life and limb. It is affirmed, without the hazard of successful challenge, that both these grave considerations were met during the late civil war, by the medical staff of the army, with a skill and patriotic devotion to duty, alike worthy the profession and the cause.

In the old army, *i. e.*, the army as it existed prior to the war of 1861–5, the Regimental Hospital was the only field-hospital recognized or provided for in the "army regulations." During the Autumn of 1861, and the Winter of 1862, this plan was still adhered to. The sick, who could not be properly treated in quarters, were, by order of the Surgeon, sent to the Regimental Hospital, which was conveniently located near, and, indeed, formed part of the camp. To provide therefor, each regiment was allowed three hospital tents, one Sibley tent, and one "A" tent. The hospital tents, each measuring 14x16 feet area measure, were usually pitched one behind another, so that they formed three communicating apartments. The other tents were used by the attendants, and also for kitchen purposes. When the capacity of this hospital became overtaxed, the surplus was sent to General Hospital in Washington. Sometimes it was expedient as well as convenient to locate the regimental hospitals in or near dwelling-houses that had been vacated by their owners or occupants, and abandoned to the tender consideration of the Union forces. Our illustration shows an example of such utilization of a deserted house for hospital conveniences by the 49th Regiment, N. Y. Volunteers. The hospital here shown was situate about half a mile in rear of the troops, on a road leading through Camp Griffin to Chain Bridge. This hospital was in operation on the spot depicted from the Autumn of 1861 to March 8, 1862. It is from an India ink sketch, drawn and presented to me by a member of Co. B, whose name I have forgotten.

In the Spring of 1862, when the army was moved to the Peninsula, and it became necessary, in order to properly mobilize it for the field,

Field Hospital at Savage's Station, Va., June, 1862, showing the reception and first care of the wounded from the battles of Ellerson's and Gaines's Mills. From a photograph in possession of the author since 1862.—W. W. P.

to reduce the baggage and camp equipage to the minimum, each regiment was allowed but one hospital tent. Depot hospitals were, however, on our arrival at the new line of operations, established at the army base, for the reception of the sick in excess of the regimental accommodations. During the siege of Yorktown, conveniences of a like character were provided at Ship Point and at Old Point Comfort. While the army was before Richmond in May and June, 1862, large field-hospitals were established at Savage's Station and at White House; and their capacity was taxed to the uttermost, in the care of the sick and wounded during that portion of the Peninsular campaign.

At Savage's Station were collected the wounded from the battles of Ellerson's and Gaines's Mills, numbering over 2,000 men.[1] In the movement to Harrison's Landing it was found impracticable to remove this hospital, and it was, therefore, left to fall into the enemy's hands. It was liberally supplied with surgeons, nurses, stores, and rations, and everything done for the comfort of its inmates which the exigencies of the service permitted. On Sunday, June 29th, at or about five o'clock P. M., the Confederates, under General Magruder, appeared in force in the vicinity of this hospital, approaching both along the railroad and the Williamsburg road. A vigorous attack was made upon a portion of the second corps, which was drawn up in line on the open field south of the hospital, facing west. A formidable railroad battery opened fire at once, the first shell exploding directly over the hospital, which was in direct range of the fire. Other shots quickly followed, with the effect of killing one man and wounding others in the hospital, destroying some of the tents, and causing much dismay among the already suffering inmates. The hospital also contained a number of Confederate wounded, among whom was Colonel Lamar, of the Eighth Georgia Regiment. A flag of truce was immediately sent out by the surgeon in charge, notifying the commanding officer of the Confederate force which made the attack on the hospital, that it was suffering from the fire of his batteries, and that some of his own men were likely to be among the victims. The following reply was returned:

The hospital will not be fired into unless undue advantage is taken of its flag.

(Signed) A. CONRAD,

*A. A. Gen'l, Confed. Forces.*

The division of General William F. (Baldy) Smith, in which I was then serving, had moved on the road toward White Oak Swamp a

1. For other illustrations of this hospital, see the *Century Magazine* for July, 1885, pp. 459-461. From the various battle-fields during the "seven days" fighting, the number in this hospital was increased to about 3,000.—W. W. P.

short time previous to the commencement of the engagement, which was known as the Battle of Savage's Station. But as soon as it was ascertained that the enemy had appeared in force and was making determined effort, General Smith countermarched his division and, on reaching the field, threw it in on the left of Sumner's Corps, where the fighting was spirited and considerable loss was sustained, chiefly by the Vermont Brigade under General Brooks. Night soon came on, however, and quickly put an end to the action, excepting some desultory firing that was continued until a later hour. Smith's wounded were collected at a small house and shop about a mile down the Williamsburg road, toward White Oak Swamp, where there was a little opening of a few acres in the woods.

About nine o'clock P. M., while I was busily engaged in caring for these wounded, Dr. J. B. Brown, Medical Director of the Sixth Corps, called me aside, stating that he had orders from General Franklin to leave the wounded where they were, with medical officers and nurses, and that I had been selected to remain behind with them. He stated, furthermore, that all our forces would pass by before midnight, moving toward White Oak Swamp, and that I had better make such arrangements, at once, as would enable me to comply with the order; whereupon my horses were despatched with the troops to avoid capture, and hastily collecting such hospital supplies as were available, I once more addressed myself to the care of the wounded. By midnight, or a little after, the retreating columns had all passed by on their way to White Oak Swamp, where the conflict was to be renewed on the morrow, with all the fierceness of its deadly energy.

The consciousness of being between the lines with the certainty of falling into the enemy's hands in the morning, together with the pressing duties of the hour, were sufficient to counteract the fatigues that otherwise would have speedily brought that much-needed repose, which I vainly sought about two o'clock in the morning. Soon after dawn the Rebel skirmishers appeared slowly advancing through the woods, coming to a halt on a line with the hospital. Some officers immediately rode up who were informed of the condition of affairs, but before the conversation was ended, General "Stonewall" Jackson himself appeared upon the scene. Upon application he ordered a guard, consisting of a sergeant and twelve men, for the purpose of protecting the hospital during the passing of his columns; and, after ascertaining the facts as to our authority for being there, gave the order for his line to advance. All day long the steady tramp of the foe made unwelcome music to our ears. They were a cheerful lot, flushed with what they delusively supposed was victory of a decisive nature; their uniforms (?)

were tattered, but their muskets were bright; and their cannon, chiefly marked "U. S.", were, for the most part, drawn with rope traces.

Some time during the forenoon the head of General D. H. Hill's division halted in front of the hospital, and from him a pass was obtained which authorized me to visit the battle-field of the evening before, for the purpose of ascertaining if any of the wounded had been overlooked. This I did in the afternoon accompanied by one of the guards, and met on the field a Confederate ambulance squad in charge of a sergeant, already engaged in the same duty. A few wounded were found in the woods on the left, and I also counted about seventy Union dead, most of which lay in the opening through which the Williamsburg road passes out into the open field.[1]

On Tuesday, July 1st, the wounded left in my care were moved up to the main hospital at Savage's Station, and distributed to its wards. The guns of Malvern Hill were distinctly heard during the entire afternoon, and the cheering news of the enemy's defeat soon reached our ears. Two weeks later a train-load of wounded, on flat cars, was moved into Richmond, and I accompanied them. We arrived late in the evening and, owing to some mismanagement in regard to the arrival of the ambulances, were compelled to remain at the station all night. Next morning the wounded were distributed to the buildings then used for hospital purposes, and the medical officers were sent to Libby prison, then also using as a hospital. I was directed to report to the commandant, Lieutenant Turner, who ordered a search of my person, ostensibly to ascertain if I had in possession any counterfeit Confederate money. Not finding any, he contented himself with seizing my pocket case of surgical instruments, which he regarded as contrabrand of war, casting a longing eye upon some gold coin which I happened fortunately to have, but which he dare not take. I was assigned to the care of Union wounded in a large tobacco warehouse on Cary street, about four blocks east of Libby, and continued upon that duty until my release, which happily occurred in a very few days.

Richmond was, at this time, one vast hospital. Every building which could possibly be made to serve the purpose was filled with wounded, either Union or Confederate. These buildings were, for the most part, tobacco warehouses, and were devoid of any of the proper conveniences pertaining to hospital service. The Union wounded lay upon bare floors with, possibly in some instances, a blanket underneath and a knapsack for a pillow. The air was hot and stifling, saturated with the sickening odor of stale tobacco, and alto-

1. See plan of the battle of Savage's Station in the *Century* for July, 1885, p. 460.—W. W. P.

gether it was a most uncomfortable state of affairs. However, I saw no disposition to treat any of our wounded with unkindness, and presumed the authorities were doing the best they could with the resources at their command.

One day, not long after entering upon duty at this hospital, acting upon the suggestion of Assistant Surgeon J. Sim Smith, U.S.A., a fellow prisoner,[1] I obtained a pass from Lieutenant Turner to visit the officers' prison on Eighteenth street, where some of my acquaintances were incarcerated whom I was desirous of seeing. This prison was also a large tobacco warehouse, and contained several hundred officers, among whom were Generals McCall and Reynolds, the former captured at the battle of Glendale, June 30th, and the latter at Gaines's Mill, June 27th. At the solicitation of my friends, Captain McLean, 5th U. S. Cavalry, and Captain Theodore B. Hamilton, 33d N. Y. Volunteers, I remained all night as their guest; and on my return to Libby next morning, to my surprise I found a train of ambulances loaded and ready to start for Aiken's Landing with wounded for exchange. I immediately applied to Dr. Cullen,[2] Longstreet's medical director, who had charge of the matter, for permission to accompany the train. This he readily granted and, mounting the nearest ambulance, I rode with the driver to Aiken's Landing on the James River, a distance of about ten miles from Richmond. Here we were delivered to the hospital steamer "Louisiana," Lieutenant-Colonel Sweitzer, of General McClellan's staff, truce officer in charge, and reached Harrison's Landing next morning in safety; having, however, anchored in the river near the point of embarkation for the night, to avoid the danger of fire from the enemy's shore batteries, as our flag would not protect us after dark. This was the first transaction of exchange under the cartel, just then concluded between the commissioners, Major-General John A. Dix for the United States, and Colonel Robert Ould for the Confederate authorities.

General McClellan boarded the "Louisiana" by steam launch soon after we anchored off Harrison's bar, and spent nearly an hour in close conversation with Major Clitz and Captain Chambless, two regular army officers wounded at Gaines's Mill, and who were lying upon cots in the saloon of the vessel. General J. E. B. Steuart, the famous Confederate trooper, paid several visits to these officers while they were quartered in Libby, sitting between their cots which were contiguous to each other, and passing a few moments of apparently pleasant conversation with them at each visit. The medical officers

1. Since deceased.—W. W. P.

2. I had met Dr. Cullen at Williamsburg in May, when he was sent into our lines by Longstreet to look after his wounded.—W. W. P.

and nurses who were fit for duty here rejoined their respective commands, and the "Louisiana" proceeded on her way with the wounded to northern hospitals.

About this time an important change took place in the administrative head of the medical department of the Army of the Potomac. Surgeon Charles S. Tripler, U. S. A., a most able and accomplished officer, who, from the accession of McClellan, had performed the duties of medical director, was nominated by the President to be medical inspector-general of the United States Army; and Surgeon Jonathan Letterman, U. S. A., was appointed to the vacancy occasioned by this promotion. Dr. Tripler's experience had been wide, and his training of such a nature as well suited him to the responsibilities of the office he had so long and admirably filled; but the difficulties to overcome had been many and various, and while the campaign just ended had taxed his energy and capacity to their uttermost, it had yet left as a heritage other and newer experiences, as well as a trained medical staff,—resources of inestimable value to be drawn upon by his successor.

These experiences had demonstrated the inadequacy of the regimental hospital system, as well as the defectiveness of the brigade hospitals which were tried for a time, to meet the necessities of military operations conducted on so large a scale as now, where the marches were so long and arduous, and the fighting so terrible and bloody. The new medical director addressed himself almost at once to the solution of the difficult problems of providing a comprehensive field hospital system, which should be adequate to the great exigencies of the military operations of so vast an army, and a disciplined ambulance service as well, which should be competent to promptly and efficiently transport the sick and wounded, both on the march and in battle. Orders were promulgated on August 24, 1862, on the subject of the ambulance corps, and on October 30, 1862, in relation to field hospitals; and so complete were the plans set forth in these orders in all their details, that they remained in force without material change, until the end of the war. Moreover, their provisions were subsequently adopted by the Surgeon-General, and made the uniform practice throughout all the armies in the field.

Briefly summarized, these plans were as follows: Each division hospital was to organize with a staff, consisting of one surgeon in charge; one assistant surgeon as recorder; one assistant surgeon to provide food and shelter; three medical officers to perform operations, each operator to have three assistants; and additional medical officers, according to necessity, to attend the wards, dress wounds, etc.

There were also one chief hospital steward, one chief cook, one ward master, and a few nurses attached to the permanent organization. Extra hospital stewards, cooks, nurses, and other attendants were to be detailed for duty as occasion required. On the march, or in camp, the extra medical officers, hospital stewards, cooks, nurses, and attendants remained with their respective regiments; only the permanent staff was constantly on duty at the hospitals, or accompanied the ambulance trains.

The ambulances were organized into division trains with a first lieutenant in command, and second lieutenants from each brigade as assistants; the entire trains of each corps being commanded by a captain attached to the corps commander's staff. A sufficient number of enlisted men were detailed from the ranks to properly man the trains of each division, in the proportion of two men and a driver to each ambulance, and a mounted sergeant from each regiment.[1] A medicine wagon, properly supplied with stimulants, dressings, and medicines for each brigade, also formed a part of the division field-hospital equipment. Each division train was provided with a saddler, a blacksmith, and a traveling forge, to keep the train in order; and each ambulance was supplied with stretchers, buckets, kettles, lanterns, beef stock, bed-sacks, and kitchen utensils.

This is but a faint setting forth of the great labor and multiform details which such a comprehensive plan involved, and, whereas in July the young medical director of the Army of the Potomac came into office finding a medical department somewhat disorganized and chaotic, by the end of October he had gathered around him an amply equipped and thoroughly drilled hospital staff, as well as a trained, organized, and efficient ambulance corps, adequate to meet the pressing necessities of the great army in its self-imposed Herculean labor.

As soon as a battle became imminent, the medical director of the corps ordered the establishment of a hospital for each division of the corps, in positions selected by himself convenient to the troops, yet sufficiently out of range of fire to insure comparative safety. Houses were, when available, chosen for these hospital sites, the adjacent grounds usually affording conveniences for pitching the tents, obtaining water, and other supplies for the comfort of the wounded. The wagons were ordered up at once, the hospital staff repaired to the site selected, and, under the superintendence of the surgeon in charge, prepared the hospital for the reception of the wounded. Tents were pitched; straw, fuel, water, blankets, etc., provided; hospital flag conspicuously hoisted; markers displayed at suitable points to indicate

1. These men wore chevrons, half-chevrons, and cap-bands of green, as distinctive badges.—W. W. P.

the route to the hospital; kitchen organized, and everything made ready for active usefulness. On the arrival of the wounded, the operating surgeons and their assistants took their places at the operating-tables in the rear of the medicine wagons, over which a fly had been spread, and where instruments, dressings, anesthetics, and stimulants were at hand.

One medical officer, usually the junior assistant surgeon, remained with each regiment, together with a nurse or two and the hospital orderly, which latter carried a field companion supplied with dressings and other necessaries; and these were ordered to establish themselves at temporary depots, at such distance in the rear of each regiment as would ensure safety to the wounded. Sometimes these temporary depots were consolidated into one or two for each brigade; especially was this plan considered more feasible when regiments were small. At these advance depots, the ambulances received the wounded for conveyance to division hospital, and, as fast as they were loaded and driven away, their places were supplied with others from the ambulance reserve, still farther in the rear. On the arrival of the ambulances at the hospital, the recorder made an entry of each case in a book provided for that purpose, stating name, rank, company, and regiment of the soldier, and the nature of the wound, together with any particulars of value to note. If an operation appeared to be required, the case was sent at once to the operating staff,—otherwise to the wards, and given in charge of a dresser. This record was further perfected to show the treatment, operation (if any), and the result or disposition of the case, daily reports therefrom being made to the medical director of the corps, and by him sent, with those of the other divisions, to the medical director of the army. And so the work went on in its busy round, until the wounded were all brought off the field, operations made, wounds dressed, patients fed, reports made up and sent in, and the wounded finally shipped to the depot hospitals at the army base. So complete was the working of this system, that, on several occasions after the severest battles, I have seen more than a thousand wounded cared for in one of these hospitals, the urgent operations made, and all the first attention rendered, within a few hours after the arrival of the first ambulance load.

These hospitals were subjected to a rigid system of inspection, both during action and at other times, not only by the medical inspectors of the corps, but also by medical inspectors from the headquarters of the army; so that it was almost impossible for affairs to go very wrong in their conduct. If, perchance, evils crept in, or inefficient officers obtained responsible places, they were of certain detection and swift remedy. The ambulance trains were also subjected to frequent and thorough inspections; the men were drilled and instructed in their duties, and everything pertaining to this important service constantly maintained at the highest possible standard of efficiency.

On the march, each division ambulance train followed immediately in the rear of the troops to which it belonged, and was accompanied by the permanent staff of the hospital, viz.: the surgeon-in-charge, the executive officer, and the recorder. When a soldier was taken sick on the march, one of his regimental medical officers gave him an ambulance pass, which entitled him to make his way slowly along, or rest by the wayside until the train came up. One of the medical officers accompanying it examined the soldier and his pass, and, if proper, gave orders for his admission to an ambulance. On reaching the camp for the night, the sick and foot-sore thus gathered up were either returned to their regiments or retained in hospital, according to the nature and severity of the cases.

At the first battle of Fredericksburg, December 13th, 1862, the hospital of the Second Division, Sixth Army Corps, where I served as recorder, was located at the Bernard mansion,[1] a large stone house situated near the south bank of the Rappahannock river, about half a mile to the left of Franklin's Crossing. The owner, a haughty Virginian of the old school, had decamped with the Confederate forces on our approach, and so hasty had been the departure that the partly filled glasses and uncorked bottles of half-drunk wine, still standing on the dining-table and open side-board, attested to the convivial nature of his last night at home. It was understood at the time that several Confederate officers, including some of high rank, were partakers of Bernard's hospitality until a late hour that night, but, warned of the approach of the Union columns, host and guests hastily departed together, leaving behind them the tell-tale evidences of the night's hilarity. The house was comfortably, even luxuriously, furnished, and several fine pictures, together with other articles of taste and refinement, gave evidence of the wealth and culture of the late occupants. The next day, Bernard returned, but his behavior was so insolent that he was placed under guard, and subsequently sent to Washington, where he was given quarters in the old Capitol Prison.

On the lawn in front of the house, where the tall trees lifted their stately forms majestically towards the heavens, were seen, during a temporary lull in the battle, a group of generals with their attendant staff-officers and orderlies. Conspicuous among the number was a young brigadier-general of cavalry, the gallant George D. Bayard. While conversing with Generals Franklin, Smith, and others, a solid shot, ricochetting

1. This house was subsequently burned, and an accurate illustration of the ruins, which I visited June, 1863, just before the Gettysburg campaign commenced, may be found in *The Century*, August, 1886, p. 637.

across the field, struck him down, and he was brought into the hospital with a mortal wound, from the effects of which he expired twenty-four hours later. He was to have been married in five days more, it was said, to one of Philadelphia's fairest daughters. When it was finally decided to withdraw to the north bank of the river, the wounded of the Sixth Corps were removed to a temporary hospital near Falmouth. As soon as cars could be obtained, they were shipped to Acquia Creek, and thence by steamers to Washington. I was selected to accompany those from the Second Division, and reached Acquia Creek with them in charge about eight o'clock in the evening. Two steamers lying at anchor in the harbor were ordered to the wharf, and the transshipment to them was accomplished by three o'clock A. M. Washington was reached at seven in the morning, but it was not until three in the afternoon that the last of the wounded were loaded into ambulances, and on their way to the General Hospital. I had eaten nothing up to this time since leaving Falmouth, nearly twenty-four hours before, but the wounded were served with hot coffee and sandwiches at the Sixth-street wharf by the Sanitary Commission agents.

When I reached the camp of the Sixth Corps, near White Oak Church, on my return from Washington, I found awaiting me a pleasant surprise in the nature of a letter from Surgeon-General S. Oakley Vander Poel, S. N. Y., promoting me to be surgeon of the 57th Regiment N. Y. Volunteers, in the First Division of the Second Army Corps.

My service with the 49th Regiment and the Sixth Corps terminated upon the issuance of the following order:

HEADQUARTERS LEFT GRAND DIVISION,
CAMP NEAR WHITE OAK CHURCH, VA.

SPECIAL ORDER, No. 35. December 27, 1862.

. . . . . . . . . . . . . . . .

5. The following named officers, having tendered their resignation, are honorably discharged from the Military Service of the United States.

Assistant Surgeon William W. Potter, 49th N. Y. Vols., to enable him to accept a commission as surgeon of the 57th N. Y. Vols.

. . . . . . . . . . . . . . . .

By Command of MAJOR-GENERAL FRANKLIN.

(Signed,) M. T. McMAHON,

*Major and A. A. A. General.*

(*To be concluded in November number.*)

## Figures and Tables

**Figure f1:**
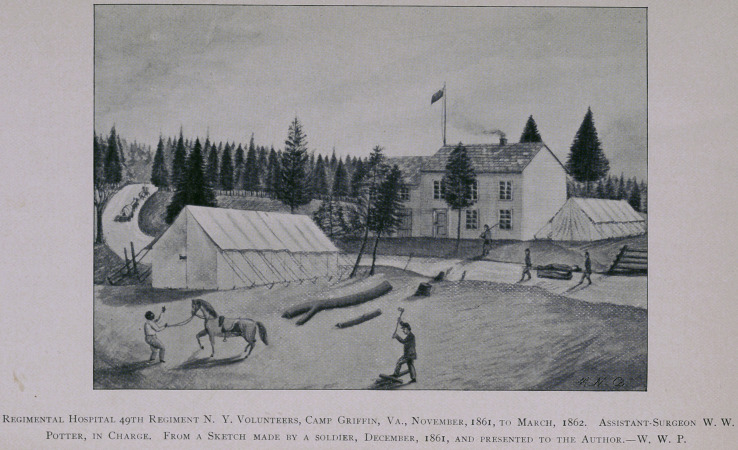


**Figure f2:**